# Machine Learning and Canine Chronic Enteropathies: A New Approach to Investigate FMT Effects

**DOI:** 10.3390/vetsci9090502

**Published:** 2022-09-13

**Authors:** Giada Innocente, Ilaria Patuzzi, Tommaso Furlanello, Barbara Di Camillo, Luca Bargelloni, Maria Cecilia Giron, Sonia Facchin, Edoardo Savarino, Mirko Azzolin, Barbara Simionati

**Affiliations:** 1 Research & Development Division, EuBiome S.r.l., 35131 Padova, Italy; 2 San Marco Veterinary Clinic and Laboratory, 35030 Veggiano, Italy; 3 Department of Information Engineering, University of Padova, 35131 Padova, Italy; 4 Department of Comparative Biomedicine and Food Science (BCA), University of Padova, 35020 Legnaro, Italy; 5 Department of Pharmacological Sciences, University of Padova, 35131 Padova, Italy; 6 Department of Surgery, Oncological and Gastrointestinal Science, University of Padova, 35121 Padova, Italy; 7 Ospedale Veterinario San Francesco, 31038 Castagnole, Italy

**Keywords:** fecal microbiota transplantation, canine chronic enteropathy, chronic diarrhea, dysbiosis, microbiome, machine learning

## Abstract

**Simple Summary:**

Fecal microbiota transplantation (FMT) represents a very promising approach to decrease disease activity in chronic enteropathies (CE). Although CE and dysbiosis are undoubtedly connected, the relationship between remission mechanisms and microbiome changes has not been elucidated yet. Indeed, CE is a heterogeneous disease consisting of many different subtypes, and the dynamic of the microbial community is very complex. The aim of this study was to report the clinical effects of oral freeze-dried FMT in dogs affected by CE, analyzing the fecal microbiome before and after FMT, and comparing the microbial composition with a healthy population. Artificial intelligence algorithms were applied to address the high complexity of microbiomes. Clinical signs of improvement were observed in three-quarters of receivers, proving the effectiveness of the treatment in the freeze-dried form. Machine learning algorithms successfully predicted healthy and diseased animal categories, using microbial compositions. Every receiver showed microbiome variation after the transplant, but there was high heterogeneity in the response. These findings are the first step for further research on a larger dataset that could identify different healing patterns of microbiome changes.

**Abstract:**

Fecal microbiota transplantation (FMT) represents a very promising approach to decreasing disease activity in canine chronic enteropathies (CE). However, the relationship between remission mechanisms and microbiome changes has not been elucidated yet. The main objective of this study was to report the clinical effects of oral freeze-dried FMT in CE dogs, comparing the fecal microbiomes of three groups: pre-FMT CE-affected dogs, post-FMT dogs, and healthy dogs. Diversity analysis, differential abundance analysis, and machine learning algorithms were applied to investigate the differences in microbiome composition between healthy and pre-FMT samples, while Canine Chronic Enteropathy Clinical Activity Index (CCECAI) changes and microbial diversity metrics were used to evaluate FMT effects. In the healthy/pre-FMT comparison, significant differences were noted in alpha and beta diversity and a list of differentially abundant taxa was identified, while machine learning algorithms predicted sample categories with 0.97 (random forest) and 0.87 (sPLS-DA) accuracy. Clinical signs of improvement were observed in 74% (20/27) of CE-affected dogs, together with a statistically significant decrease in CCECAI (median value from 5 to 2 median). Alpha and beta diversity variations between pre- and post-FMT were observed for each receiver, with a high heterogeneity in the response. This highlighted the necessity for further research on a larger dataset that could identify different healing patterns of microbiome changes.

## 1. Introduction

The gut microbiota is defined as the ecological community of all commensal microorganisms that colonize the enteric environment and relate to the host in a symbiotic manner [1]. It includes archaea, bacteria, fungi, and viruses. The bacterial population is considered to be the domain with the greatest impact on intestinal balance and health. The five phyla to which almost all bacterial species in the dog gut microbiota belong are *Firmicutes*, *Fusobacteria*, *Bacteroidetes*, *Proteobacteria,* and *Actinobacteria* [2], but at the fecal level, the most represented bacterial phyla in dogs are *Fusobacteria*, *Bacteroidetes,* and *Firmicutes* [3,4].

One of the main symptoms for which dog owners turn to the veterinarian is the onset of diarrhea (alone or with other gastrointestinal symptoms, such as vomiting), a challenging problem for both the animal and its owner.

One of the most frequent factors underlying the manifestation of diarrhea in adult dogs is chronic enteropathy (CE), a chronic inflammation that can affect parts of the intestinal tracts. The etiopathogenesis of this condition is still not entirely clear, so it is classified according to the response to the therapy into food-responsive enteropathy (FRE), antibiotic-responsive enteropathy (ARE), immunosuppressant-responsive enteropathy (IRE), and non-responsive enteropathy (NRE) [5]. Regardless of the cause, what generally characterizes all forms of CE is gut dysbiosis, with a reduction in fecal bacterial diversity [6]. Since a healthy gut microbial flora tends to inhibit or to contrast any enteric inflammatory conditions [7], one major goal of therapeutic approaches to CEs is to modulate the gut microbiota. This can be performed through either a dietary approach, or the use of pre-/pro-biotics and symbiotics (often in combination with other therapies), antibiotic therapy, or fecal microbiota transplantation (FMT).

FMT involves transferring fecal material from a healthy donor to the gastroenteric tract of a recipient with a disease, with the aim of restoring healthy intestinal microbiota. There are several ways to deliver the fecal material: via the endoscopic route, through a rectal enema or a nasogastric tube, or oral capsules containing lyophilized fecal matter. FMT has received special attention in veterinary medicine in recent years, due to multiple pieces of scientific evidence of efficacy reported in human medicine in the treatment of *Clostridioides difficile* infection [8] and many other potential applications in a variety of extra-intestinal conditions associated with intestinal dysbiosis [9,10].

To date, however, there is limited evidence of efficacy and safety regarding the administration of FMT in dogs, mainly in the form of case reports or case series [11,12,13,14,15,16,17,18,19,20,21]. The routes of administration most used by the various authors are rectal enema [14,15,18,19] and endoscopy [11,12,16,17]. Only one case report described the effectiveness of FMT carried out by using capsules containing freeze-dried fecal material [21], although this appears to be the easiest route of administration from a practical standpoint.

In this study, we analyzed the first set of data collected within the “Pet FMT Project”, a crowdsourced project that aims to understand the pathogenesis and to identify the microbial markers of CE, a complex and poorly understood disease, through the collection of thousands of fecal transplant cases in cats and dogs. Here, we present the evidence emerging from the first 56 cases of dogs affected by CE, 27 of which received clinical evaluation before and after FMT administration.

We started by characterizing the differences between healthy and CE canine gut microbiota by means of alpha and beta diversity, differential abundance analysis, and machine learning approaches. Moreover, we identified a core microbiota describing healthy microbiomes and selected a list of key features capable of capturing the main differences between healthy and diseased dogs. Secondly, we evaluated the effect of FMT performed on CE dogs by administering capsules of freeze-dried fecal material. The outcome of the FMT treatment was evaluated by the analysis of the clinical parameters and the gut microbial composition. In particular, changes in the canine chronic enteropathy clinical activity index (CCECAI) [22] were used to determine the clinical response since this index is adopted in the literature to evaluate canine enteropathies, even if a recent study [23] questioned the relationship between the value of CCECAI and the severity of the disease. Indeed, this index is calculated from the sums of sub-scores that describe clinical characteristics with different severity (e.g., vomiting, itching, loss of vitality, diarrhea), which are deemed equivalent. In any case, it is the only universally accepted metric that allows a standardized and objective evaluation of chronic enteropathies, based on clinical symptoms.

## 2. Materials and Methods

### 2.1. Study Design

This is an interventional study on privately owned dogs conducted within the “Pet FMT Project” (www.progettopetfmt.it, accessed in 30 June 2022). The owners were informed of the purposes of the study and signed an informed consent form. The study received the official approval of the Animal Welfare Committee of the University of Padova (OPBA Prot. n. 369551, on 31 July 2020).

In the present research, we analyzed the first 56 cases that met the inclusion criteria in the project (see below). All patients received FMT treatment in oral capsule form. The fecal material used for the transplant was obtained from one single healthy donor. Fecal samples for microbiome analysis were collected by the patient’s owners before the first FMT capsule administration (pre-FMT samples) and approximately 15 days after the end of FMT treatment (post-FMT samples).

Referring veterinarians evaluated CCECAI at the same time point of fecal sample collection. The clinical response was defined as a CCECAI decrease of at least two points 15 days after the end of FMT treatment, and statistical significance was determined using Wilcoxon signed rank test (two-tailed tests, *p* < 0.05 for each hypothesis).

#### Study Populations

Microbiome data for healthy controls consisted of V3-V4 16S rRNA sequencing data from 94 healthy dogs coming from a previously published work [21], and a set of data produced in the present study from four healthy dogs. Literature data were integrated after checking that extraction and sequencing protocols were the same as those used here. To further control for batch effects, a principal coordinate analysis (PCoA) on the Bray–Curtis distance was performed, showing that the internal samples (four donors) were not distinct from the 94 samples integrated from the literature (Figure S1).

The diseased population included 56 patients with CE at baseline (intention-to-treat pre-FMT samples). All client-owned dogs with CE enrolled in the study were examined by the referring veterinarian. Patients were included if they presented with a minimum of a three-week history of vomiting, diarrhea, anorexia, or weight loss. Exclusion of known digestive and extra-digestive causes of chronic GI signs was performed by the clinician requesting the FMT. Treatments based on antibiotics and probiotic administration were suspended, respectively, two or five days prior to FMT treatment.

Sixteen patients were excluded due to a lack of owner compliance, change in diet, or administration of antibiotics during the FMT treatment. Forty patients completed the treatment and were eligible for fecal sample collection 15 days after the end of the treatment (ITT post-FMT samples).

Twenty-seven patients received the CCECAI evaluation both before and after the FMT (per-protocol pre-FMT and post-FMT samples).

Figure S2 illustrates the analysis carried out to highlight the differences between the populations.

### 2.2. Donor Dogs Selection

The donor dogs were classified in good health after undergoing physical and clinical examinations (aged between 1 and 6 years, no travel history outside the local area, no history of chronic disease, allergies, immune-mediated disease, no history of vomiting, diarrhea, or treatment with antibiotics in the last 3 months, no overweight or underweight status, normal fecal conditions, no behavioral issue). The donor dogs were further examined for the following: complete blood count, serum biochemical analysis, intestinal function test (serum cobalamin and folate concentration), pancreatic enzymatic immunoassays (pancreatic lipase immunoreactivity, trypsin-like immunoreactivity), endocrine test (serum cortisol), negative for parasitic eggs on fecal floatation, negative for parasites (*Giardia, Cryptosporidium*, *Ancylostoma*, *Ascarididae*, *Trichuridae*) on ELISA fecal test (Fecal Dx^®^, IDEXX), negative for pathogenic infectious agents or toxins (Canine Enteric Coronaviruses, Canine Parvovirus-2, Canine distemper, Canine circovirus, *C. perfringens* enterotoxin, C. *perfringens* netE/netF genes, *C. difficile* toxin A and toxin B, *C. coli*, *C. jejuni*) evaluated with real-time PCR analysis (Canine Diarrhea PCR Profile Plus Panel, IDEXX), and negative for pathogenic microbes (*Salmonella* spp., thermophilic Campylobacters., hemolytic *E. coli*, coagulase-positive Staphylococci, *Klebsiella* spp., *Proteus* spp., *Yersinia* spp.) evaluated with cultural methods (Diarrhea Profile C Panel, IDEXX). Additional requirements were yearly vaccinations against leptospirosis, vaccination every three years against canine distemper, parvovirus, adenovirus, and rabies, treatment with anti-filarial and antiparasitic drugs in spring and summer, deworming every six months, and a balanced diet.

The donor was under the control of the referral veterinarian for monitoring throughout the entire donation and fully rescreened yearly, while parasites, enteropathogens, and toxins were evaluated every 90 days (Fecal Dx^®^, Canine Diarrhea PCR Profile Plus Panel, Diarrhea Profile C Panel, IDEXX).

### 2.3. FMT Preparation and Administration

We collected donor feces from a 4-year-old, intact, male, Welsh Corgi Cardigan dog. Fecal samples were collected by the donor’s owner into sterile containers and frozen at −20 °C within an hour. The frozen samples were transported weekly to the laboratory to be immediately processed.

Freeze-dried fecal microbiota were prepared following a protocol previously described, with few modifications [24]. Briefly, fecal material was homogenized with saline solution (1:3) in a blender bag to remove larger particles. The filtrate was amended with 5% trehalose and freeze-dried for 72 h. Donated material was quarantined at −80 °C until 16S rRNA sequence characterization was performed to evaluate alpha-diversity and microbial composition, and the donor’s fresh fecal samples were rescreened for parasites, pathogenic microbes, and toxins. The quarantined freeze-dried material was mixed with a single fecal lot. Each batch of capsules was prepared from a single fecal lot using size 1 (200 mg) or size 4 (100 mg) capsules (DRcaps, Capsugel). Capsules were manually filled and stored at −80 °C until needed.

Once removed from the freezer, a 1 g silica gel canister was added to the container and the capsules were delivered to patient homes at room temperature. The patient’s owner administered one capsule daily to the dog for a month. For dog body weight > 10 kg, the dosage was 200 mg daily, whereas for dog body weight ≤ 10 kg, the dosage was 100 mg daily.

### 2.4. Microbiome Analysis

Immediately after defecation, 0.1 g of feces was collected with a sterile swab and placed in a DNA collection tube (eNAT, COPAN Diagnostics Inc, Murrieta, CA, US).

Cell lysis was performed on 250 μL of the diluted fecal sample or 100 mg of FMT preparation (QIAamp PowerFecal DNA Kit, QIAGEN). Total DNA was extracted from 200 mL of the lysate, using Cador Pathogen 96 QIAcube HT Kit (QIAGEN), following the manufacturer’s instructions. Total DNA was resuspended in 100 μL of nuclease-free water and stored at −20 °C until preparation for sequencing.

16S rRNA gene was amplified by using a standard protocol and modified primers [21].

### 2.5. Bioinformatic Analysis

#### 2.5.1. Pre-Processing

Forward and reverse reads were pre-processed and merged using the Quantitative Insights into Microbial Ecology pipeline (QIIME2, version 2020.8) [25]. First, primer sequence removal was performed by means of cutadapt [26] considering no indels (insertion–deletion), an error rate equal to 0 and an overlap of 10 nucleotides, and allowing wildcard read matching (--p-no-indels; --p-error-rate 0; --p-overlap 10; --p-match-read-wildcards). The reads in which no adapter sequence was found were discarded (--p-discard-untrimmed). Then, the amplicon sequence variant (ASV) table was obtained by means of a de novo clustering procedure using the DADA2 [27] bioinformatic tool plugin. The taxonomic assignment of each ASV was determined using the Greengenes database [28] (version 13_8) and two naive Bayes classifiers—one for the healthy samples and one for the remaining samples—that were trained on the target region selected for the present study, considering the different primer pairs adopted in this study with respect to Scarsella et al. [29].

#### 2.5.2. Diversity Analysis

Alpha indices, i.e., richness (observed ASVs), Pielou, Shannon, and Faith’s phylogenetic diversity, were calculated for microbial community diversity analysis applying a rarefaction level equal to 20,000. This cut-off was chosen after verification (by means of a rarefaction plot (Figure S3)) that all the samples presented an adequate sequencing depth, and that the chosen threshold that was placed after each rarefaction curve had reached its plateau. Comparisons were made to investigate differences in alpha diversity values between healthy and CE-affected dogs (labeled as “diseased” in the following) and pre- and post-FMT samples. In detail, a *t*-test and a Wilcoxon signed-rank test were performed for healthy/diseased and pre-/post-FMT (paired) comparisons.

In order to include both phylogenetic and non-phylogenetic and both binary and abundance-based metrics, four different beta measures were also included in the study, i.e., Bray–Curtis, Jaccard, and weighted and unweighted UniFrac metrics. The resulting matrices were used for unsupervised machine learning analysis, i.e., ordination analysis with the PCoA technique, in order to investigate a possible separation of the above-mentioned groups based on their microbial composition. A PERMANOVA analysis was also run to support graphical results.

Alpha diversity analysis was performed via QIIME2 dedicated plugins, while beta diversity calculation and ordination plot production were performed in *R* (version 4.1.0) using *phyloseq* (version 1.36.0) and *vegan* (version 2.5–7) packages. For the latter task, data were previously normalized using the *GMPR* tool [30] (version 0.1.3) to allow for a robust comparison between samples.

#### 2.5.3. Differential Abundance Analysis

To identify differentially abundant (DA) features between healthy and pre-FMT samples, we selected three of the best performing and recent microbiome-specific tools, i.e., *ALDEx2* [31] (version 1.24.0), *MaAsLin2* [32] (version 1.6.0), and *ANCOMBC* [33] (version 1.2.2), and considered as the final DA features list the intersection of the results obtained from the three tools.

#### 2.5.4. Supervised Machine Learning

Two different supervised machine learning approaches were used within this study: a random forest (RF) and a sparse partial least squares-discriminant analysis (sPLS-DA). The analyses were performed in *R* by using the *randomForest* [34] (version 4.6–14) and *mixOmics* [35] (version 6.16.1) packages, with a proportional sampling of training and test set covering 65% and 35% of the total samples, respectively. As the latter approach inherently needs more data than RF to achieve its best performances, the analysis for sPLS-DA was also run with a 65–35% dataset partitioning.

#### 2.5.5. Key Features Identification

Results coming from the differential abundance analysis, random forest, and sPLS-DA approaches were intersected to identify a list of key features robust to different analysis frameworks. In detail, for both species and genus levels, we considered the intersection of (a) the list of DA taxa; (b) the list of the 30 taxa with the highest mean decrease accuracy value calculated running RF algorithm; and (c) the list of the 30 taxa with the highest loading (absolute) value coming from sPLS-DA analysis.

The resulting consensus list was then compared with the core taxa (at the species level) and the taxa (species and genus levels) included in the qPCR-based Dysbiosis Index (DI) specifically developed by Alshawaqfeh and colleagues [36] to assess microbial changes in the microbiome of dogs affected by CE.

Additionally, a set of core species defining a healthy microbiome was identified, characterized as those species that were present in more than 90% of the samples coming from healthy dogs. In addition, in this case, the analysis was only performed for healthy/diseased and pre-/post-FMT comparisons, where a sufficient sample size was available.

## 3. Results

### 3.1. Microbiome Sequence Data

The sequencing of the samples collected in this study (96 receivers and 4 donors) produced an amount of 8,904,044 raw reads, with a mean value per sample of 89,040.44 and a standard deviation (SD) of 24,857.28. After primer removal, filtering, denoising, merging, and chimera removal steps, a total of 4,899,827 reads were retained (mean: 48,998.27; SD: 18,177.24), equivalent to the 55.03% of input reads (mean: 55.06%; SD: 13.40%).

Sequencing data from a previous study (94 healthy dogs) amounted to 17,360,757 raw reads, with a mean value per sample of 184,688.90 and a standard deviation (SD) of 58,158.88. After primer removal, filtering, denoising, merging, and chimera removal steps, a total of 8,261,522 reads were retained (mean: 87,888.53; SD:25,142.48), equivalent to the 47.59% of input reads (mean: 49.39%; SD: 12.19%).

### 3.2. Analysis of Diseased vs. Healthy Population

**Figure 1:**
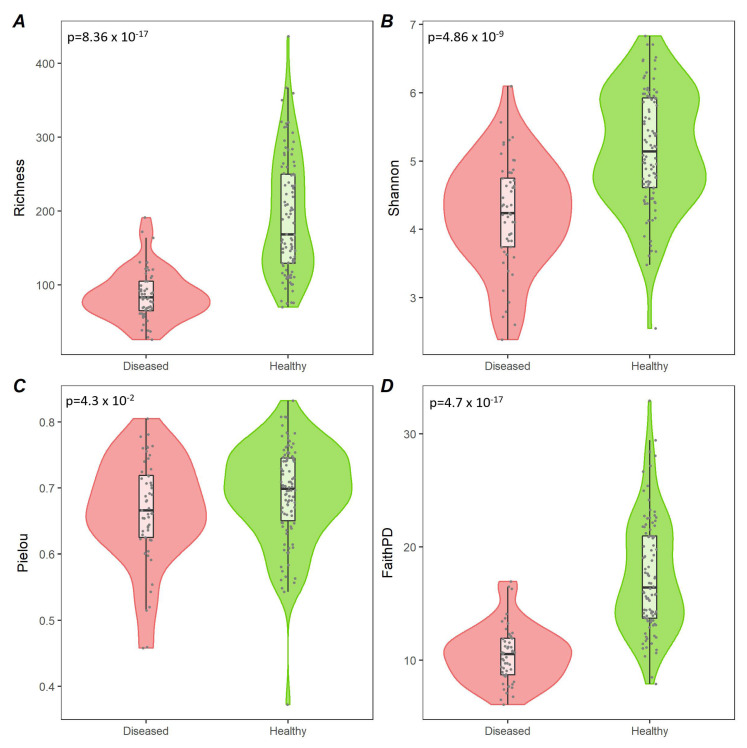
Alpha diversity comparison between healthy and diseased dogs. Violin plots representing alpha values in healthy (green) and diseased (red) groups according to richness (**A**), Shannon (**B**), Pielou (**C**), and Faith’s (**D**) phylogenetic diversity measures. The related *p*-values are visible in the top-left corner of each panel.

We compared four alpha diversity indices (richness, Shannon, Pielou, and Faith’s PD) between diseased and healthy populations. The comparison involved 53 diseased samples (three samples were discarded as the sequencing depth was below the rarefaction level) and 98 healthy samples. For all the included indices, the diseased values resulted significantly lower than the healthy ones, as shown in Figure 1.

**Figure 2:**
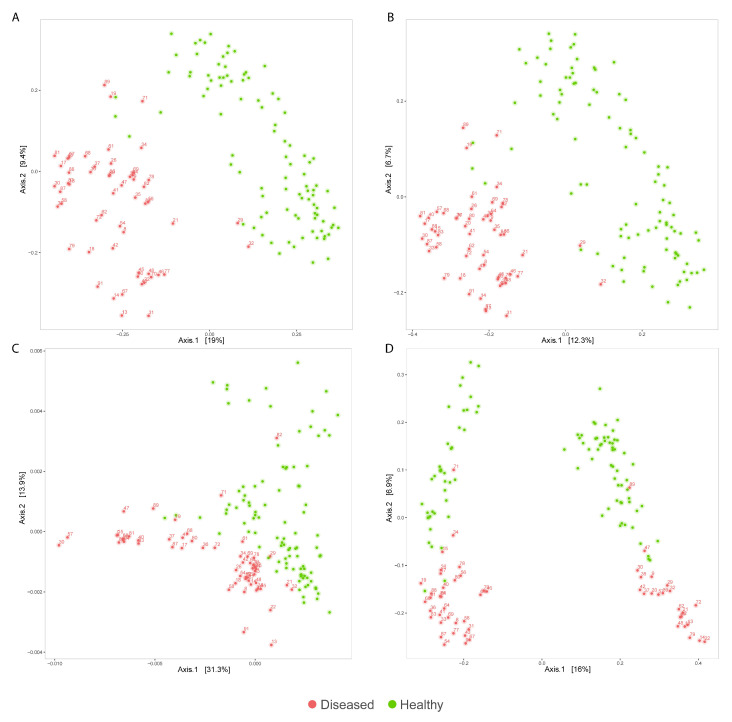
Principal coordinate analysis (PCoA) plots representing the microbial composition of healthy (green) and diseased (red) groups, calculated using Bray-Curtis (**A**), Jaccard (**B**), weighted UniFrac (**C**), and unweighted UniFrac (**D**) diversity measures.

Qualitative (unweighted UniFrac, Jaccard) and quantitative (weighted UniFrac, Bray–Curtis) evaluations of beta-diversity were also performed. All the ordination plots displayed differences in the composition between healthy dogs and CE dogs before treatment (PERMANOVA, *p* < 0.001) (Figure 2). The unweighted UniFrac diversity measure separated healthy and CE dogs on Axis2, but at the same time showed two groups, each comprising both healthy and CE dogs, separated on Axis1. This plot suggests that some low-abundance, phylogenetically distant ASVs create a separation also within healthy subjects’ microbiomes, a separation that is no more visible when including the abundance (quantitative) information, as seen in Figure 2C. An analogous situation was previously reported by Wong and her team [37], which showed how unweighted UniFrac could lead to spurious group separation due to sequencing depth constraints inherently present in 16S rRNA-gene sequencing studies.

**Figure 3:**
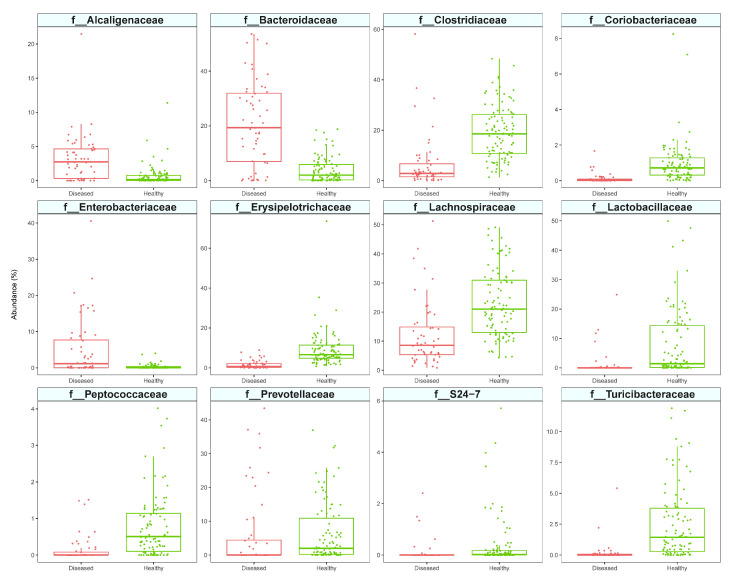
Differentially abundant families between healthy (green) and chronic enteropathies (CE) affected (red) dogs.

Twelve bacterial families were determined to be differentially abundant between the healthy population and the CE dogs with three different tools (*ALDEx2*, *MaAsLin2, ANCOMBC)* (Figure S4). CE patients had a significantly lower abundance in members of Coriobacteriaceae, Prevotellaceae, S24-7 family (current name Muribaculaceae), Lactobacillaceae, Turicibacteriaceae, Clostridiaceae, Lachnospiraceae, Peptococcaceae, and Erysipelotrichaceae, and a higher abundance in members of Bacteroidaceae, Alcaligenaceae, and Enterobacteriaceae (Figure 3). From these data, it is clear that the CE population was mainly characterized by the low abundance of some bacterial taxa rather than their excess. These findings also applied to lower taxonomic level. Indeed, only 3/25 differential abundant genera (*Bacteroides* spp., *Sutterella* spp., and unknown genus belonging to Enterobacteriaceae, Figure S5) and 2/30 differential abundant species (*Bacteroides uniformis* and an unidentified species belonging to *Roseburia* spp., Figure S6) were overrepresented in CE dogs.

To assess the predictive potential of the microbiome according to the considered variables (see Materials and Methods), we performed supervised machine learning by means of random forest and sPLS-DA approaches. This analysis was performed considering it as a two-class problem. After a random selection of a training set including 65% of our healthy/diseased or pre-FMT/post-FMT samples, we tested the fitted model on the remaining samples (35%), used as a test set.

The random forest algorithm incorrectly predicted only one subject in the test set (20 diseased, 34 healthy) (Table 1), thus meaning the model obtained a 0.98 accuracy (95% CI: (0.9011, 0.9995)), with a sensitivity value of 1 and a specificity value of 0.97 (Table S1). Cohen’s Kappa was 0.96. The list of genera and species with the related mean decrease accuracy and Gini indices values are reported in Tables S2 and S3.

**Figure 4:**
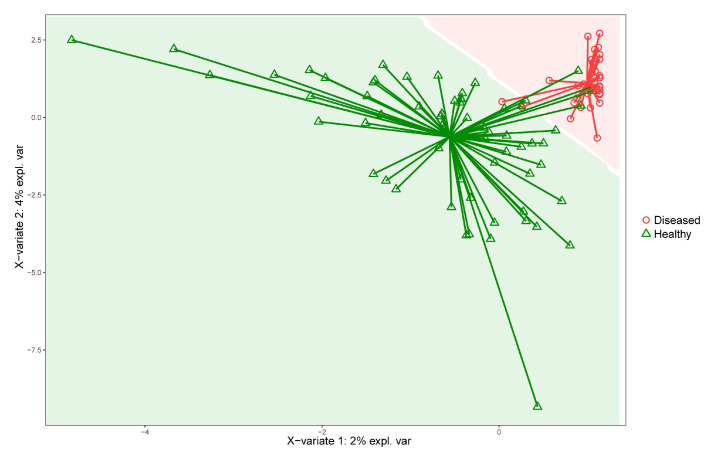
Sample prediction area plot from the sPLS-DA model applied at species level (training set: 65%; test set: 35%). Samples are classified into two classes: diseased (red) and healthy (green). To visualize the prediction area, the plot background was colored according to the predicted classification area.

The sPLS-DA results confirmed the marked difference between healthy and CE microbiomes, as shown in Figure 4. The model obtained a 0.87 accuracy (95% CI: (0.751, 0.9463)), with a sensitivity value of 0.95 and a specificity value of 0.82. The Cohen’s Kappa was 0.76.

As a final step of our analysis, we were interested in selecting a core set of species characterizing the healthy population, and in identifying those features (species and genera) that robustly distinguished healthy and enteropathic microbiomes.

The intersection of the DA features, the most important features identified by the mean decrease accuracy index calculated within the RF analysis, and the list of features of highest loadings score coming from the sPLS-DA analysis provided us with the final lists of the most relevant species and genera. These lists were then compared with the core species list and the set of species/genera included in the Dysbiosis Index for chronic inflammatory enteropathy identification. The results of this integration are shown in Table 2 and Table 3, where all the above-mentioned information was merged for the easiest multi-point evaluation. In particular, most of the taxa included in the species-level list also belong to the core species list (bold italics in Table 2) and five taxa included in the PCR-based Dysbiosis Index (in green in Table 2 and Table 3) were also selected as key features by our models. There were two missing taxa with respect to the original DI taxa, *Streptococcus* spp. and *E. coli*. As regards the last species, it is well known [38,39] that *Escherichia*, *Shigella*, *Citrobacter*, and *Salmonella* have low levels of divergence among their 16S rRNA genes and this often causes sequences coming from these bacteria to be taxonomically identified only up to their family level, i.e., Enterobacteriaceae. It is then important to highlight that, both at the species and genus level, a bacterial group belonging to Enterobacteriaceae (in orange in Table 2 and Table 3) was selected by all the three approaches (DA, RF, and sPLS-DA) among the most relevant features.

### 3.3. Effect of the FMT on Clinical Response and Microbiota

The clinical response was assessed 15 days after the end of the treatment in 27 patients as a variation in CCECAI. A positive response based on CCECAI was noted in 20/27 (74%) CE dogs after FMT. From a median value of 5 (range: 0–11) in pre-FMT samples, the CCECAI of participants decreased to 2 (range: 0–11) in post-FMT samples.

Wilcoxon signed-rank tests were applied for pairwise comparisons of CCECAI before and 15 days after the end of the treatment, considering as a sign of clinical response a decrease of CCECAI of at least 2 points. The observed difference between the pre-FMT and post-FMT CCECAI values was statistically significant (*p* = 0.0209). We then analyzed the effect of the FMT on changes in alpha and in beta diversity using the Wilcoxon signed-rank test or PERMANOVA, respectively. The level of significance was set to *p* < 0.05. Sign tests for all alpha-diversity indices were not significant, even after excluding individuals with CCECAI variation lower than 1. Overall, post-FMT samples did not differ from pre-FMT samples (Bray–Curtis *p* = 0.987; Jaccard *p* = 0.853; weighted UniFrac *p* = 0.874; unweighted UniFrac *p* = 0.985) and remained clearly distinct from healthy controls, both qualitatively and quantitatively (Figure S7).

## 4. Discussion

To our knowledge, this is the first work assessing the efficacy of FMT administered in freeze-dried capsules to a large number of dogs with CE. Additionally, we carried out a comparison at the microbiome level between a relatively large set of CE-affected dogs and 100 healthy controls, successfully integrating novel and literature data, and this provided us with several novel insights into the microbiome difference between healthy and CE-affected dogs.

Regarding microbiota analysis, alpha-diversity was significantly higher in healthy samples than in pre-FMT samples, according to all of the applied diversity indices (richness, Shannon, Pielou, Faith’s phylogenetic diversity). These results had also been observed in previous experimental investigations [6,23,40,41,42,43], even though other studies did not find significant differences in alpha diversity between healthy controls and dogs with ARE [44], SRE [45], IBD [46], and CE [47].

Likewise, beta diversity analysis showed significant differences in microbiome composition between CE and healthy dogs, suggesting the existence of microbial taxa that discriminate between the two populations.

The large number of samples analyzed within this work allowed us to identify microbial markers that are indicative of disease. First, we used three of the most relevant tools for DA features’ identification. Despite the existence of many tools expressly designed to perform this kind of analysis on 16S rRNA-gene sequencing data, none of these is currently univocally considered as outperforming compared to the others. The conclusion drawn by the most recent literature comparing various differential abundance tools is that the best practice would be to consider more than one method and to use a consensus approach to obtain robust biological conclusions [48]. Our analysis showed that the majority of DA taxa identified between healthy and CE-diseased samples were overrepresented in healthy dogs, while only a few taxa were more abundant in CE dogs. The latter represent candidate markers of dysbiosis and include the family Enterobacteriaceae, a hallmark of canine enteropathies in almost all studies, and the family Alcaligenaceae, which in dogs are mainly represented by *Sutterella* spp., an overabundant genus in the acute hemorrhagic diarrhea [43], and the Bacteroidaceae family, which includes both beneficial species and opportunistic pathogens.

Second, we were able to run both unsupervised and supervised machine learning techniques on our data. Machine learning has already been used to discriminate healthy dogs from dogs with IBD, a form of CE, in a previous work [49], where RF was applied and a list of the 20 most relevant species was reported. While in the present work this analysis was performed at the species level, Vazquez–Baeza and colleagues [49] based their analysis on OTUs. Consequently, in [49], some taxonomic assignments among 20 relevant species were repeated, and the list was finally reduced to 13 unique species. Seven out of thirteen species are included in the list published here (Table S3), while the remaining 6 belong to *Streptococcus*, *[Ruminococcus]*, Clostridiaceae, Lachnospiraceae, Ruminococcaceae, and Clostridiales. Classification discrepancies at the family or order level could be attributed to the taxonomic assignment made from OTUs instead of ASVs. In fact, the first approach increases the possibility that the assignment occurs at higher taxonomic levels. As regards the genus *Streptococcus*, in a recent meta-analysis [50], it was shown that it is the only DI taxa that did not discriminate between healthy dogs and dogs with gastrointestinal disorders. Since the diseased population in Vazquez–Baeza and colleagues [49] was affected by IBD and not by a generic chronic enteropathy, one might postulate that *Streptococcus* spp. may play a more important role in IBD than other gastrointestinal diseases.

In the list of the most relevant features identified with sPLS-DA there were two taxa, *Peptococcus* sp. and *Roseburia* sp., which were not present in the lists obtained with RF, showing how the use of multiple algorithms can lead to more comprehensive results.

The information produced in DA and machine learning analyses was merged together and compared to the identified core species list and the DI taxa. This integrated analysis led us to identify a list of key features (species and genera) that robustly describe the difference between healthy and enteropathic microbiomes and to show a great overlap between these relevant features, the healthy core microbiota, and the taxa included in the well-established PCR-based DI. Indeed, 18/26 core microbiome species were also present also in the list of the key features, with their median abundance being lower in CE dogs. This result remarks that, despite the great diversity of clinical cases here considered, the dysbiotic microbiome is mainly characterized by the lack of beneficial species, and their combined abundance is used by machine learning algorithms to discriminate CE individuals from healthy populations. Although dysbiosis is a frequent feature of canine CEs, a universal pattern has not been identified so far [51]. Several studies showed that dysbiotic microbiome profiles have a higher heterogeneity than healthy microbiomes, this effect is proposed by the “Anna Karenina principle” [52] and could explain why only a few species are globally overrepresented in patients. Deficiency or absence of key species disrupts microbiome balance, leading to the proliferation of several other species, which varies from case to case. This statement does not imply the absence of CE subgroups characterized by the presence of specific strains or by the excess of certain bacterial groups, but a large number of samples is needed to identify them. The relevance of the key features is confirmed by the fact that several species and genera listed in Table 2 and Table 3 were also identified as differentially abundant in dogs with gastrointestinal disease in previous works, either based on qPCR assays or 16S rRNA gene-based sequencing [11,40,41,43,47,49].

On the clinical side, the 27 CE patients receiving FMT capsules daily for a month showed an improvement of the CCECAI score of three points in the median, with 22/27 (74%) patients that were in remission or responded to FMT according to CCECAI variation. These results are consistent with the data presented in a congress abstract [53], where clinical improvement was noted in 24/33 (72%) CE dogs after FMT. Patient characteristics were similar to the ones included in this study, with a median Canine IBD Activity Index (CIBDAI) of 5 (range: 2–17), which decreased significantly to a median CIBDAI of 2 (range: 1–9) in the month after the last FMT. A previous interventional study [18] showed that the median CCECAI decreased significantly when FMT was used to treat a population of dogs with IBD. These findings are in agreement with our results, even if in that study the fecal material was administered endoscopically. Our data underlined that oral administration can be effective while ensuring an easy delivery method for both patients and owners. FMT administered in the form of freeze-dried capsules also proved to be effective and convenient in a recent human trial [54]. The lack of a CE control population not receiving FMT is a limitation of this study, although the disease history with patients reporting symptoms of enteritis for a median of 2 years suggests that a spontaneous improvement in our population of diseased dogs is unlikely. Another possible limitation of the present study could be the relatively short period of antibiotic treatment suspension preceding FMT treatment. However, this was the largest amount of time we could ask the patients’ owners to leave their pets without antibiotics. Indeed, the participation in this project worked on a voluntary basis and all the involved dogs suffered from CE symptoms; therefore, it was not acceptable to ask the owners to extend the suspension time with a high probability of exposing their pets to a new onset of gastroenteric symptoms.

While alpha diversity is a good marker of a healthy/diseased state, no significant difference was found in pre-/post-FMT comparison in any of the considered indices. In beta-diversity plots, no grouping was observed when looking at pre-/post-FMT variables, with all the samples being mixed together and still separable from the healthy ones. However, when looking in detail at individual changes after FMT, receivers generally showed a shift from their pre-FMT microbiota, but changes varied in the type of response to the treatment. This agrees with the heterogeneity of the response to FMT treatment observed in single patients, and suggests that future studies on bigger datasets could bring more knowledge on FMT.

Since alpha and beta diversity are inadequate as healing biomarkers, a greater sample size would allow the application of differential abundance and machine learning techniques to a less generic grouping of patients. To date, taxa associated with FRE, NRE, and IBD have been identified [47,55]; however, the classification based on the therapy response, rather than on the origin of the disease or clinical data could be a confounding factor.

To increase the number of patients, we are currently collecting data within an open-source, citizen-science project called the “Pet FMT Project” (www.progettopetfmt.it, accessed in 30 June 2022). We believe that our approach is promising in defining a new mapping of the microbiome, with clinical usefulness for the discrimination and the monitoring of dogs affected by CE. A future challenge is the collection of objective metadata necessary to reveal new insights into complex healing mechanisms.

## Figures and Tables

**Table 1:** Confusion matrix obtained by running the random forest (RF) classification model estimated at species level on a test set of 54 subjects.

		Reference	
		CE	Healthy
Prediction	CE	20	1
	Healthy	0	33

**Table 2:** List of the key species differentiating between healthy and CE microbiomes. The species were selected by intersecting the relevant species list coming from differential abundance (DA), RF, and sPLS-DA analyses. Only the species that were present in at least two out of three of these lists were included in this table, where the last column shows the number of methods that identified them as relevant (see Materials and Methods). Species in bold italic are taxa belonging to the core species list (see Results), while the green and orange colored ones are taxa included in or related to the Dysbiosis Index. Taxonomies ending with “s__” mean that there was the best match in Greengenes, but that best match did not have an assignment at the species level. In contrast, a “__” is used to indicate that classification did not arrive down to the species level because a close match did not exist in the reference database or because multiple equivalent matches were available.

Species	Methods
k__Bacteria;p__Actinobacteria;c__Coriobacteriia;o__Coriobacteriales;f__Coriobacteriaceae;g__Adlercreutzia;s__	3
k__Bacteria;p__Actinobacteria;c__Coriobacteriia;o__Coriobacteriales;f__Coriobacteriaceae;g__Collinsella;s__	3
***k__Bacteria;p__Actinobacteria;c__Coriobacteriia;o__Coriobacteriales;f__Coriobacteriaceae;g__Slackia;s__***	3
k__Bacteria;p__Bacteroidetes;c__Bacteroidia;o__Bacteroidales;f__Bacteroidaceae;g__Bacteroides;s__uniformis	3
***k__Bacteria;p__Firmicutes;c__Bacilli;o__Turicibacterales;f__Turicibacteraceae;g__Turicibacter;s__***	3
k__Bacteria;p__Firmicutes;c__Clostridia;o__Clostridiales;f__[Mogibacteriaceae];g__;s__	3
* **k__Bacteria;p__Firmicutes;c__Clostridia;o__Clostridiales;f__Clostridiaceae;g__Clostridium;s__hiranonis** *	3
***k__Bacteria;p__Firmicutes;c__Clostridia;o__Clostridiales;f__Lachnospiraceae;g__Blautia;s__***	3
***k__Bacteria;p__Firmicutes;c__Clostridia;o__Clostridiales;f__Lachnospiraceae;g__Blautia;s__producta***	3
***k__Bacteria;p__Firmicutes;c__Clostridia;o__Clostridiales;f__Lachnospiraceae;g__Dorea;s__***	3
***k__Bacteria;p__Firmicutes;c__Erysipelotrichi;o__Erysipelotrichales;f__Erysipelotrichaceae;g__;s__***	3
***k__Bacteria;p__Firmicutes;c__Erysipelotrichi;o__Erysipelotrichales;f__Erysipelotrichaceae;g__Allobaculum;s__***	3
***k__Bacteria;p__Firmicutes;c__Erysipelotrichi;o__Erysipelotrichales;f__Erysipelotrichaceae;g__Clostridium;s__spiroforme***	3
k__Bacteria;p__Firmicutes;c__Erysipelotrichi;o__Erysipelotrichales;f__Erysipelotrichaceae;g__Coprobacillus;s__	3
k__Bacteria;p__Bacteroidetes;c__Bacteroidia;o__Bacteroidales;f__[Paraprevotellaceae];g__;s__	2
***k__Bacteria;p__Bacteroidetes;c__Bacteroidia;o__Bacteroidales;f__[Paraprevotellaceae];g__[Prevotella];s__***	2
***k__Bacteria;p__Bacteroidetes;c__Bacteroidia;o__Bacteroidales;f__Bacteroidaceae;g__Bacteroides;s__***	2
***k__Bacteria;p__Bacteroidetes;c__Bacteroidia;o__Bacteroidales;f__Prevotellaceae;g__Prevotella;s__copri***	2
k__Bacteria;p__Firmicutes;c__Bacilli;o__Lactobacillales;f__Lactobacillaceae;g__Lactobacillus;s__	2
***k__Bacteria;p__Firmicutes;c__Clostridia;o__Clostridiales;f__Lachnospiraceae;g__[Ruminococcus];__***	2
***k__Bacteria;p__Firmicutes;c__Clostridia;o__Clostridiales;f__Lachnospiraceae;g__[Ruminococcus];s__***	2
k__Bacteria;p__Firmicutes;c__Clostridia;o__Clostridiales;f__Lachnospiraceae;g__Roseburia;s__	2
k__Bacteria;p__Firmicutes;c__Clostridia;o__Clostridiales;f__Peptococcaceae;g__Peptococcus;s__	2
***k__Bacteria;p__Firmicutes;c__Clostridia;o__Clostridiales;f__Peptostreptococcaceae;g__;s__***	2
k__Bacteria;p__Firmicutes;c__Erysipelotrichi;o__Erysipelotrichales;f__Erysipelotrichaceae;g__[Eubacterium];s__	2
***k__Bacteria;p__Firmicutes;c__Erysipelotrichi;o__Erysipelotrichales;f__Erysipelotrichaceae;g__[Eubacterium];s__biforme***	2
***k__Bacteria;p__Firmicutes;c__Erysipelotrichi;o__Erysipelotrichales;f__Erysipelotrichaceae;g__Catenibacterium;s__***	2
***k__Bacteria;p__Fusobacteria;c__Fusobacteriia;o__Fusobacteriales;f__Fusobacteriaceae;__;__***	2
k__Bacteria;p__Proteobacteria;c__Betaproteobacteria;o__Burkholderiales;f__Alcaligenaceae;g__Sutterella;s__	2
k__Bacteria;p__Proteobacteria;c__Gammaproteobacteria;o__Aeromonadales;f__Succinivibrionaceae;g__Anaerobiospirillum;s__	2
k__Bacteria;p__Proteobacteria;c__Gammaproteobacteria;o__Enterobacteriales;f__Enterobacteriaceae;__;__	2

**Table 3:** List of the key genera differentiating between healthy and diseased microbiomes. The genera were selected by intersecting the relevant genera list coming from DA, RF, and sPLS-DA analyses. Only the genera that were present in more than two out of three of these lists were included in this table, where the last column shows the number of methods that identified them as relevant (see Materials and Methods). Genera colored in green and orange are taxa respectively included in or related to the Dysbiosis Index (see Materials and Methods). Taxonomies ending with “s__” mean that there was a best match in Greengenes, but that the best match did not have an assignment at the species level. In contrast, a “__” is used to indicate that classification did not arrive down to the species level because a close match did not exist in the reference database or because multiple equivalent matches were available.

Genera	Methods
k__Bacteria;p__Actinobacteria;c__Coriobacteriia;o__Coriobacteriales;f__Coriobacteriaceae;g__Adlercreutzia	3
k__Bacteria;p__Actinobacteria;c__Coriobacteriia;o__Coriobacteriales;f__Coriobacteriaceae;g__Slackia	3
k__Bacteria;p__Bacteroidetes;c__Bacteroidia;o__Bacteroidales;f__Bacteroidaceae;g__Bacteroides	3
k__Bacteria;p__Firmicutes;c__Bacilli;o__Lactobacillales;f__Lactobacillaceae;g__Lactobacillus	3
k__Bacteria;p__Firmicutes;c__Bacilli;o__Turicibacterales;f__Turicibacteraceae;g__Turicibacter	3
k__Bacteria;p__Firmicutes;c__Clostridia;o__Clostridiales;f__Clostridiaceae;g__Clostridium	3
k__Bacteria;p__Firmicutes;c__Clostridia;o__Clostridiales;f__Lachnospiraceae;g__Blautia	3
k__Bacteria;p__Firmicutes;c__Clostridia;o__Clostridiales;f__Lachnospiraceae;g__Dorea	3
k__Bacteria;p__Firmicutes;c__Clostridia;o__Clostridiales;f__Peptococcaceae;g__Peptococcus	3
k__Bacteria;p__Firmicutes;c__Erysipelotrichi;o__Erysipelotrichales;f__Erysipelotrichaceae;g__Allobaculum	3
k__Bacteria;p__Firmicutes;c__Erysipelotrichi;o__Erysipelotrichales;f__Erysipelotrichaceae;g__Catenibacterium	3
k__Bacteria;p__Firmicutes;c__Erysipelotrichi;o__Erysipelotrichales;f__Erysipelotrichaceae;g__Clostridium	3
k__Bacteria;p__Firmicutes;c__Erysipelotrichi;o__Erysipelotrichales;f__Erysipelotrichaceae;g__Coprobacillus	3
k__Bacteria;p__Proteobacteria;c__Betaproteobacteria;o__Burkholderiales;f__Alcaligenaceae;g__Sutterella	3
k__Bacteria;p__Proteobacteria;c__Gammaproteobacteria;o__Enterobacteriales;f__Enterobacteriaceae;__	3
k__Bacteria;p__Actinobacteria;c__Coriobacteriia;o__Coriobacteriales;f__Coriobacteriaceae;g__Collinsella	2
k__Bacteria;p__Bacteroidetes;c__Bacteroidia;o__Bacteroidales;f__Prevotellaceae;g__Prevotella	2
k__Bacteria;p__Firmicutes;c__Clostridia;o__Clostridiales;__;__	2
k__Bacteria;p__Firmicutes;c__Clostridia;o__Clostridiales;f__[Mogibacteriaceae];g__	2
k__Bacteria;p__Firmicutes;c__Clostridia;o__Clostridiales;f__Lachnospiraceae;g__Roseburia	2
k__Bacteria;p__Firmicutes;c__Clostridia;o__Clostridiales;f__Peptostreptococcaceae;g__	2
k__Bacteria;p__Firmicutes;c__Clostridia;o__Clostridiales;f__Ruminococcaceae;g__Faecalibacterium	2
k__Bacteria;p__Firmicutes;c__Erysipelotrichi;o__Erysipelotrichales;f__Erysipelotrichaceae;g__	2
k__Bacteria;p__Firmicutes;c__Erysipelotrichi;o__Erysipelotrichales;f__Erysipelotrichaceae;g__p-75-a5	2
k__Bacteria;p__Fusobacteria;c__Fusobacteriia;o__Fusobacteriales;f__Fusobacteriaceae;__	2
k__Bacteria;p__Fusobacteria;c__Fusobacteriia;o__Fusobacteriales;f__Fusobacteriaceae;g__Fusobacterium	2
k__Bacteria;p__Proteobacteria;c__Gammaproteobacteria;o__Aeromonadales;f__Succinivibrionaceae;g__Anaerobiospirillum	2

## Data Availability

Data available on request from the authors.

## Supplementary Materials

The following supporting information can be downloaded at: https://www.mdpi.com/article/10.3390/vetsci9090502/s1, Figure S1: Intention-to-treat samples beta diversity plot; Figure S2: Experimental design; Figure S3: Rarefaction plot; Figure S4: Venn diagram; Figure S5: Differentially abundant species; Figure S6: Differentially abundant genera; Figure S7: Per-protocol samples beta diversity plot; Table S1: Random forest (RF) performance metrics; Table S2: Mean decrease accuracy and Gini indices values at genus level; Table S3: Mean decrease accuracy and Gini indices values at species level.
