# Supplementary figures and images for: Machine Learning and Canine Chronic Enteropathies: A New Approach to Investigate FMT Effects
Source: Vet Sci. 2022 Sep 13;9(9):502. doi: 10.3390/vetsci9090502 (PMC9505216; doi:10.3390/vetsci9090502)

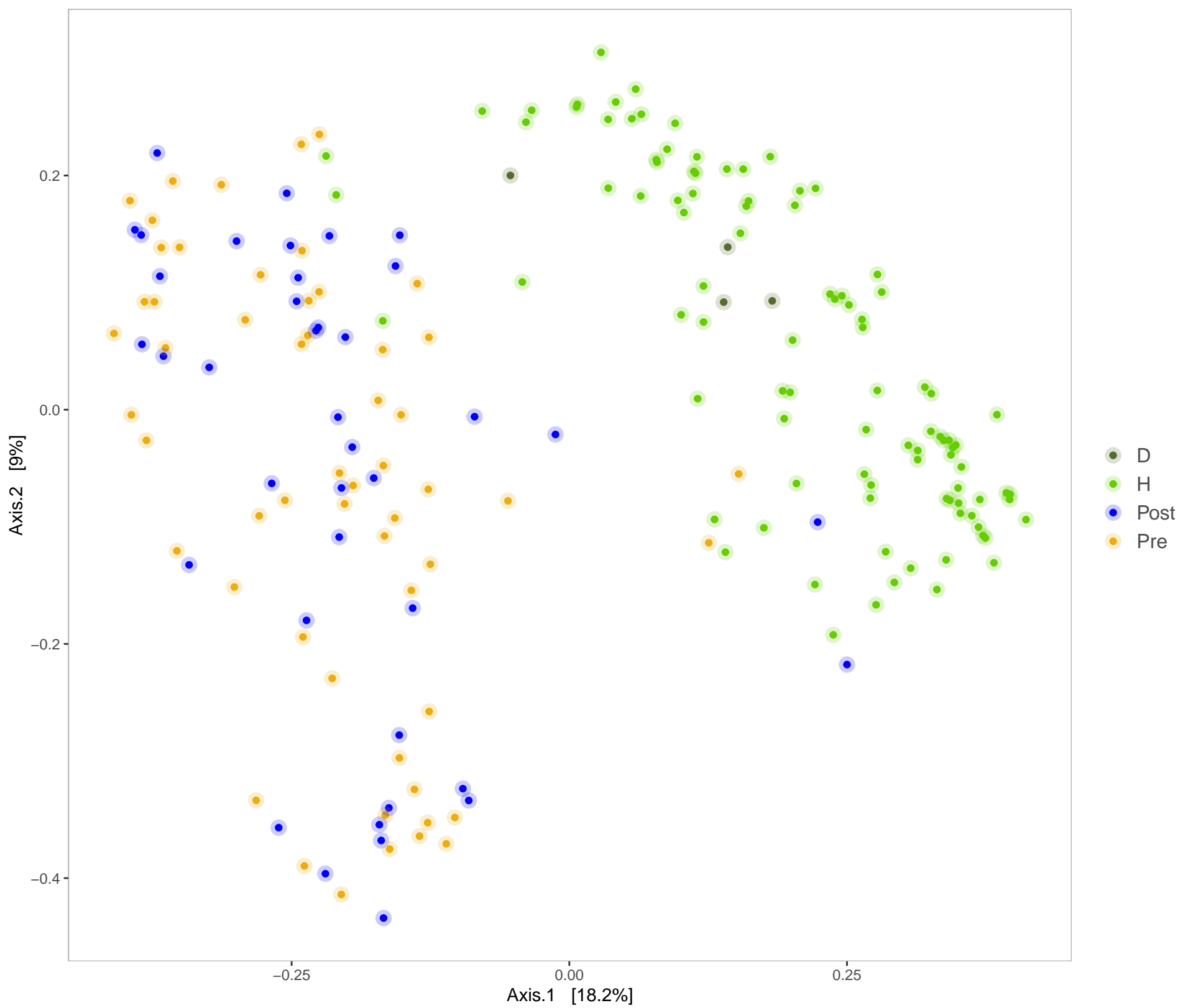

Supplement: Supplementary file 1 [file vetsci-09-00502-s001.zip › a-Figure_S1.pdf]

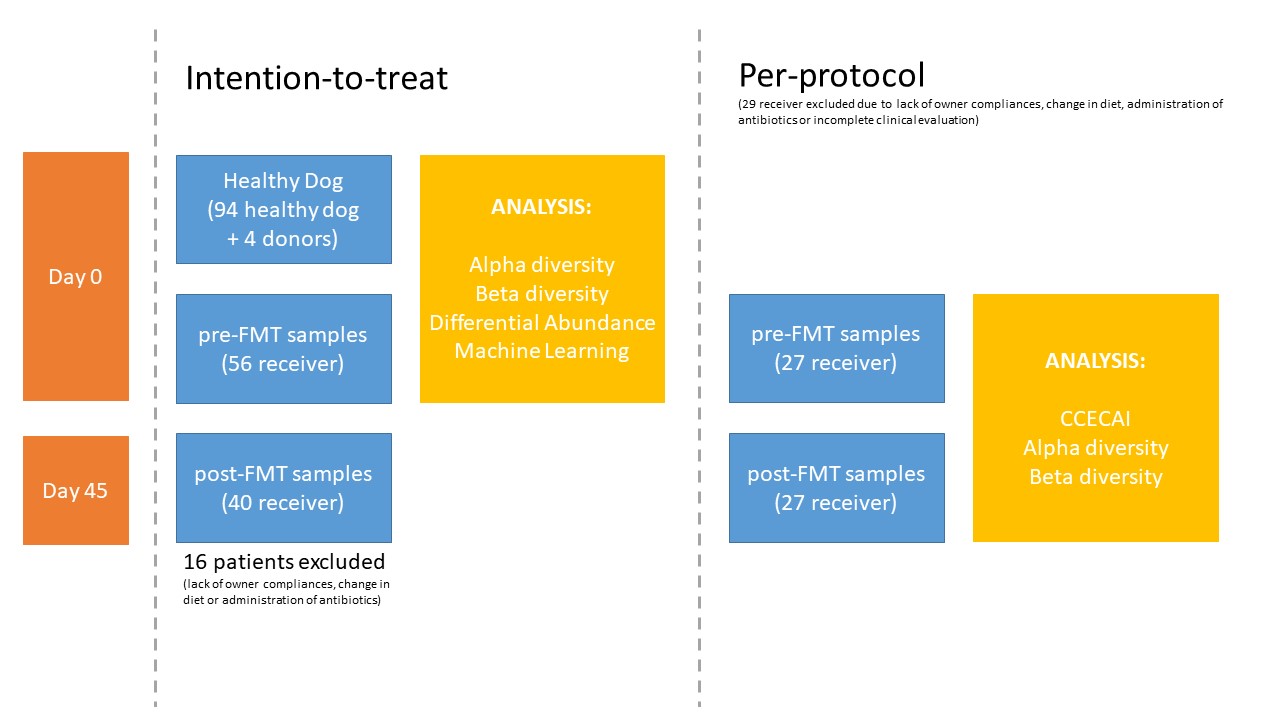

Supplement: Supplementary file 1 [file vetsci-09-00502-s001.zip › a-Figure_S2.jpg]

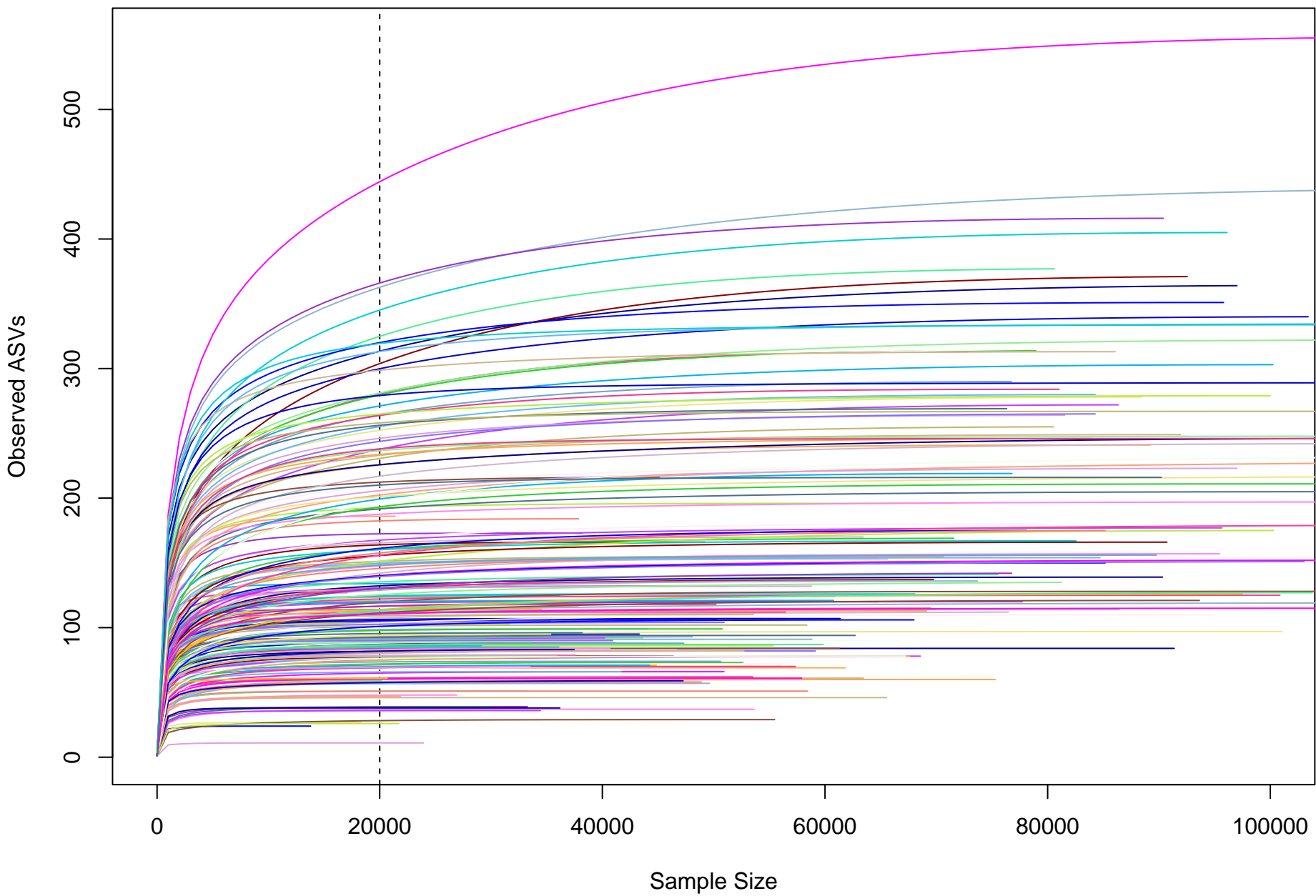

Supplement: Supplementary file 1 [file vetsci-09-00502-s001.zip › a-Figure_S3.pdf]

Aldex2

ANCOMBC

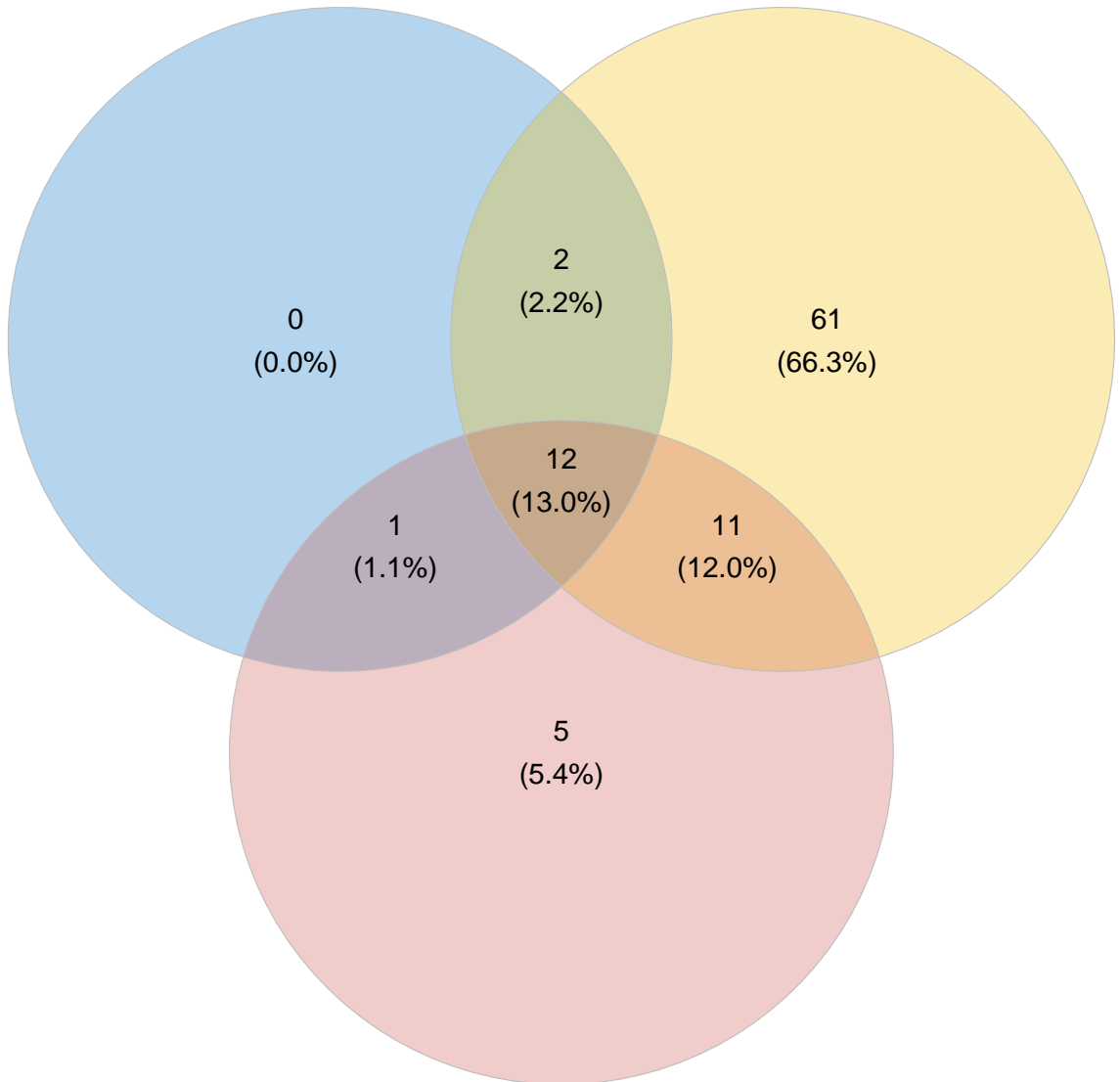

MaAsLin2

Supplement: Supplementary file 1 [file vetsci-09-00502-s001.zip › a-Figure_S4.pdf]

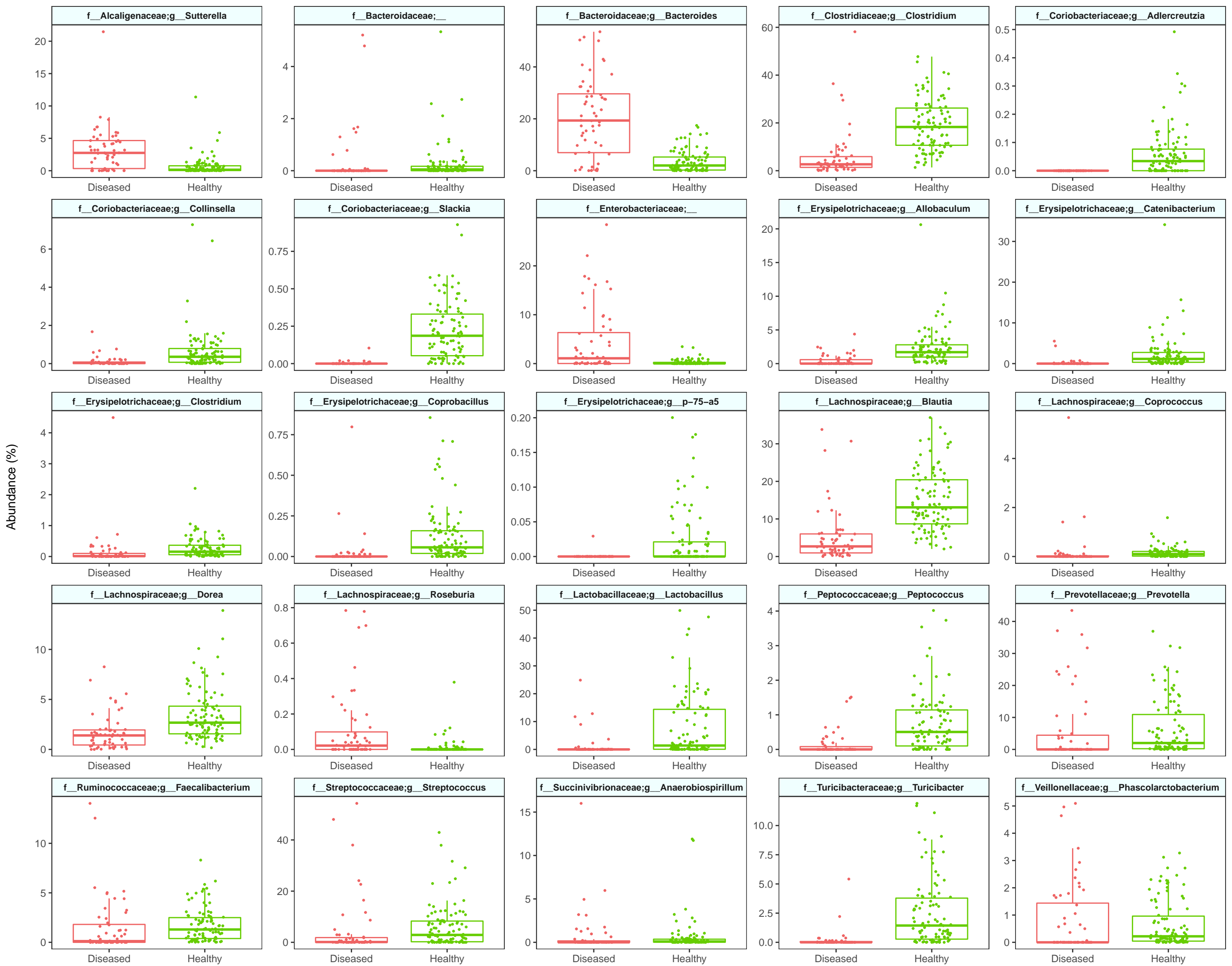

Supplement: Supplementary file 1 [file vetsci-09-00502-s001.zip › a-Figure_S5.pdf]

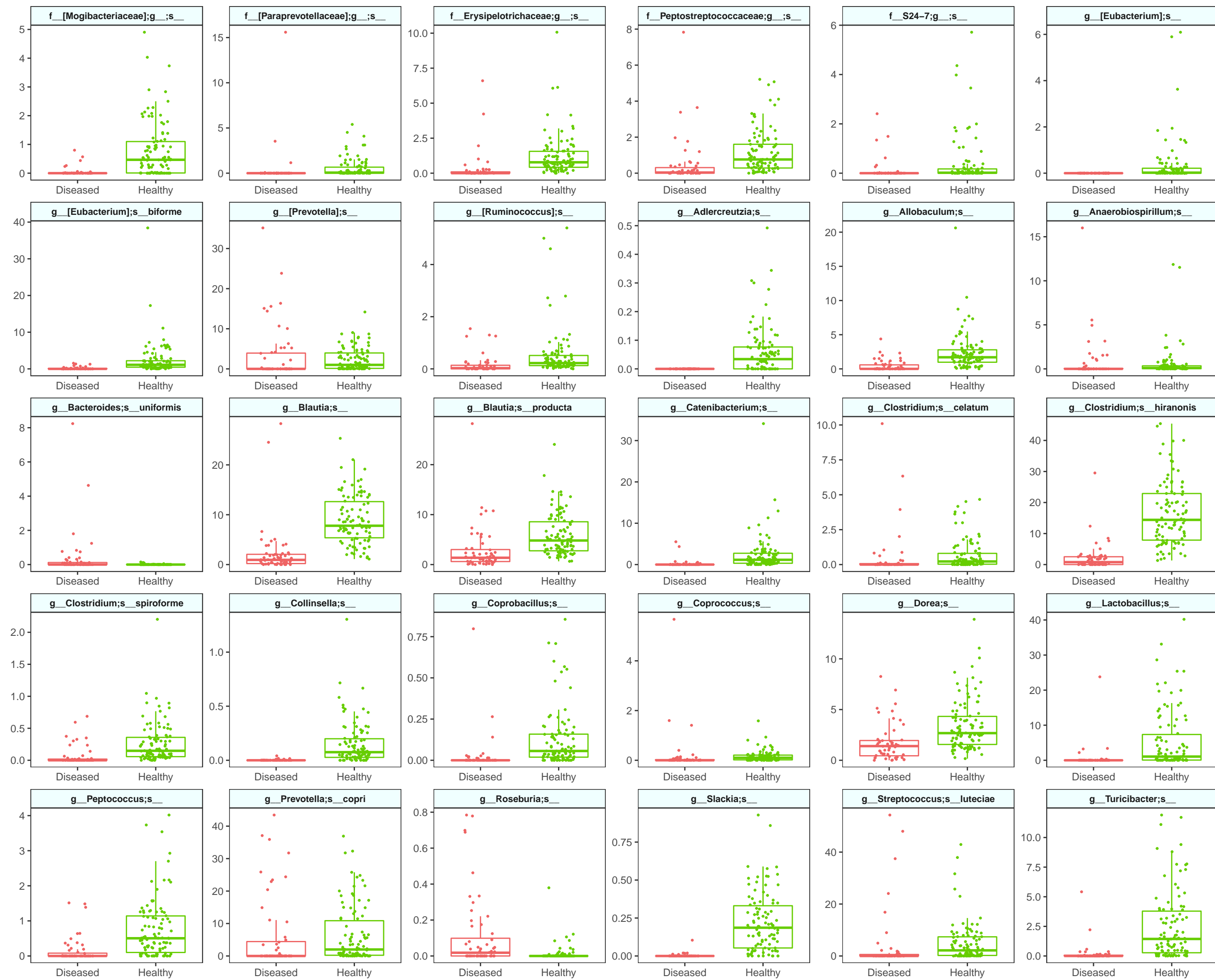

Supplement: Supplementary file 1 [file vetsci-09-00502-s001.zip › a-Figure_S6.pdf]

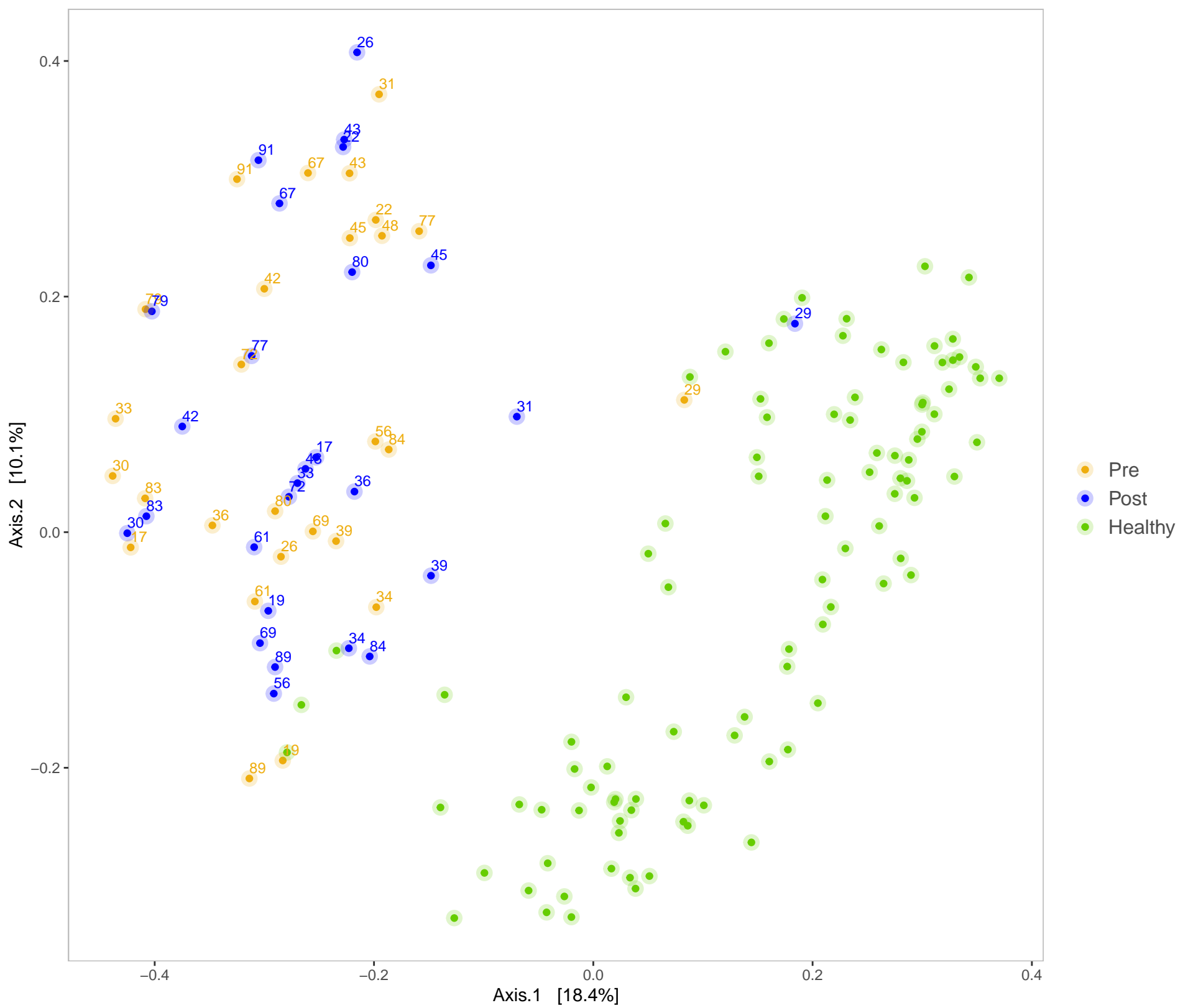

Supplement: Supplementary file 1 [file vetsci-09-00502-s001.zip › a-Figure_S7.pdf]
